# 305. PBMC-Derived Transcriptomic Signatures Accurately Discriminate Between Viral, Bacterial, and Fungal Infections and can be Translated to Real-World Human Infections

**DOI:** 10.1093/ofid/ofad500.377

**Published:** 2023-11-27

**Authors:** Julie M Steinbrink, Yiling Liu, Ricardo Henao, Ephraim L Tsalik, Geoffrey S Ginsburg, Elizabeth Ramsburg, Christopher W Woods, Micah T McClain

**Affiliations:** Duke University, Durham, North Carolina; Duke University, Durham, North Carolina; King Abdullah University of Science and Technology, Thuwal, Makkah, Saudi Arabia; Duke University, Durham, North Carolina; Duke University, Durham, North Carolina; Spark Therapeutics, Philadelphia, Pennsylvania; Duke University, Durham, North Carolina; Duke University, Durham, North Carolina

## Abstract

**Background:**

Analysis of host gene expression patterns (‘signatures’) can provide diagnostic information to determine the etiology of acute infection. *In vitro* experiments can be used to supplement our knowledge about these signatures.

**Methods:**

We performed *in vitro* human peripheral blood mononuclear cell (PBMC) challenges with influenza virus, gram-negative (*Escherichia coli*) and gram-positive (*Streptococcus pneumoniae*) bacteria, and fungal pathogens (*Candida albicans, Cryptococcus neoformans and gattii*). Exposed human cells were analyzed for differential gene expression utilizing Affymetrix microarrays. Logistic regression models were applied to the gene expression data to identify signatures capable of identifying each infection etiology. PBMC-derived signatures for each pathogen class were then applied to a microarray dataset of peripheral blood samples from human patients with candidemia.

**Results:**

A 41-gene multinomial classifier was developed which correctly classified viral, bacterial, or fungal infection with 94-98% accuracy. Gene expression patterns in PBMC challenges show substantial overlap with gene expression changes in natural infection. For fungal infection, 31 of the top 50 differentially expressed genes (DEGs) from the PBMC challenge overlapped with responses seen in patients with candidemia. Twenty of these genes were upregulated, including those involved in IgG binding (FCGR1A and FCGR1B). A subset of these genes were able to accurately identify human cases of candidemia in an independent dataset with an area under the receiver operating characteristic curve (auROC) of 0.94. Pathway analysis of differentially expressed genes revealed pathogen class-specific immune responses. For fungal infections in particular, a marked upregulation of inflammatory responses was seen including cellular responses to stress, eosinophil chemotaxis (CCL24, SOCS1/2) and T cell proliferation and migration (IL2, CXCL9).

**Conclusion:**

*In vitro* PBMC challenges can support ability to generate host transcriptomic signatures able to discriminate between bacterial, viral, and fungal infection, and their performance can be translated to real-world human infections.

**Disclosures:**

**Julie M. Steinbrink, MD, MHS**, Duke University: Methods to Detect and Treat a Fungal Infection **Ricardo Henao, PhD**, Duke University: Methods to Diagnose and Treat Acute Respiratory Infections **Ephraim L. Tsalik, MD, PhD**, Danaher Diagnostics: VP and Chief Scientific Officer for Infectious Disease|Duke University: Methods to Diagnose and Treat Acute Respiratory Infections; Methods to Detect and Treat a Fungal Infection **Geoffrey S. Ginsburg, MD, PhD**, Duke University: Methods to Diagnose and Treat Acute Respiratory Infections **Elizabeth Ramsburg, PhD**, Spark Therapeutics: Head Of Central Nervous System & Research **Christopher W. Woods, MD, MPH**, Biomeme Inc: Methods to diagnose and treat acute respiratory infections|Biomeme Inc: Chief Medical Consultant|Biomeme Inc: Ownership Interest **Micah T. McClain, MD, PhD**, Biomeme Inc: Methods to diagnose and treat acute respiratory infections

